# Compensatory and Dissociative Coping in Young Adults: A Person-Centered Analysis of Their Associations with Childhood Maltreatment and Problematic Pornography Use

**DOI:** 10.1007/s10508-026-03495-1

**Published:** 2026-08-17

**Authors:** Ori Meir Komemi, Yaniv Efrati

**Affiliations:** Faculty of Education, Bar-Ilan University, Ramat Gan, 5290002 Israel

**Keywords:** Compensatory–dissociative behavior, Problematic pornography use, Childhood maltreatment, Latent profile analysis, Escapism

## Abstract

Building on the Compensatory-Dissociative Online Gaming model (Giardina et al., 2024a), the current study proposes a compensatory–dissociative continuum of online pornography consumption. Using a person-centered approach, we identified distinct young adult profiles based on compensatory and dissociative patterns. We first examined differences in psychopathological symptoms, impulsivity, loneliness, emotion regulation, basic psychological needs, and early childhood trauma. We then explored how early childhood trauma moderates the links between compensatory and dissociative patterns profiles, problematic pornography use characterized by loss of control, distress, and impairment, and overall online pornography consumption. A total of 608 young adults (436 men [73%], 165 women [27%]), ranging in age from 18 to 27 years (M = 24.3, SD = 4.8) completed self-report measures assessing compensatory–dissociative pornography use, early childhood trauma, psychopathological symptoms, impulsivity, loneliness, difficulties in emotion regulation, satisfaction of basic psychological needs, and both problematic and overall pornography use. Latent profile analysis identified three profiles (Low, Mid, and High) based on compensatory and dissociative tendencies. Participants in the High profile group reported the most severe psychological outcomes, whereas those in the Low profile group demonstrated the healthiest functioning. Childhood sexual abuse severity was significantly linked to greater problematic pornography use and higher frequency of pornography consumption. Unexpectedly, participants in the Mid profile group, not the High profile group, reported the highest online pornography consumption. Results highlight the value of person-centered approaches in revealing complex trauma related behavioral patterns and emphasize the importance of tailored interventions addressing problematic pornography use among young adults.

**Supplementary Information:**

The online version contains supplementary material available at https://doi.org/10.1007/s10508-026-03495-1.

## Introduction

The concept of escapism has become increasingly important for understanding problematic pornography use (Bőthe, et al., 2021). However, it remains poorly defined, which has led to inconsistent findings and difficulties in comparing results across studies (Bőthe et al., 2021, 2024a; Fernandez & Griffiths, 2021; Testa et al., 2024). Recently, a critical review of compensatory and dissociative processes in problematic online gaming led to the development of the Compensatory-Dissociative Online Gaming (C-DOG) model, clarifying distinctions between related constructs such as escapism, immersion, and avoidance (Giardina et al., 2024a). Central to this model is the distinction between escapism and escape. Escapism refers to a temporary, psychologically driven shift away from a challenging physical reality into a virtual space, whereas escape reflects a more enduring rejection of reality (Calleja, 2010; Coulson et al., 2020). When applied to pornography use, this perspective suggests that users primarily undergo a psychological shift rather than a physical one, such as mentally disengaging from stress or negative emotions while remaining physically present in their everyday environment (Giardina et al., 2024a).

Recent conceptualizations suggest positioning escapism within a broader motivational framework for pornography consumption, highlighting its distinct role compared to traditional consumption motives such as exploration or mastery (Bőthe et al., 2021; Kor et al., 2022). Specifically, Giardina et al. (2024a) propose viewing escapism not simply as another motive, but as a broader coping process that emerges in response to unmet psychological needs (Pal & Arpnikanondt, 2024; Wang et al., 2024). Thus, escapism differs from general immersion, which reflects simple engagement with the activity. It also differs from escape, which involves a more persistent detachment from and rejection of external reality. Adopting this nuanced perspective, online pornographic environments should not be framed in opposition to real life but understood as distinct from physical spaces and embedded within everyday experiences (Giardina et al., 2024a). Such spaces may offer individuals emotional simulation opportunities similar to dreams, allowing for the experimental and prospective processing of emotional experiences and the exploration of psychological needs.

To clarify the theoretical grounding of the present study, we briefly introduce the C-DOG (Giardina et al., 2024a) model and its adaptation to pornography use. The C-DOG model conceptualizes engagement with virtual activities along a continuum from compensatory to dissociative involvement, defined by the degree of integration between physical and virtual environments. At the compensatory pole, virtual engagement maintains a flexible and homeostatic relationship with the physical world, supporting temporary emotion regulation and need fulfillment while preserving adaptive return to offline life. At the dissociative pole, by contrast, virtual and physical environments become rigidly separated, with virtual engagement serving primarily to protect the self from overwhelming affect and chronic need frustration.

**Fig. 1:**
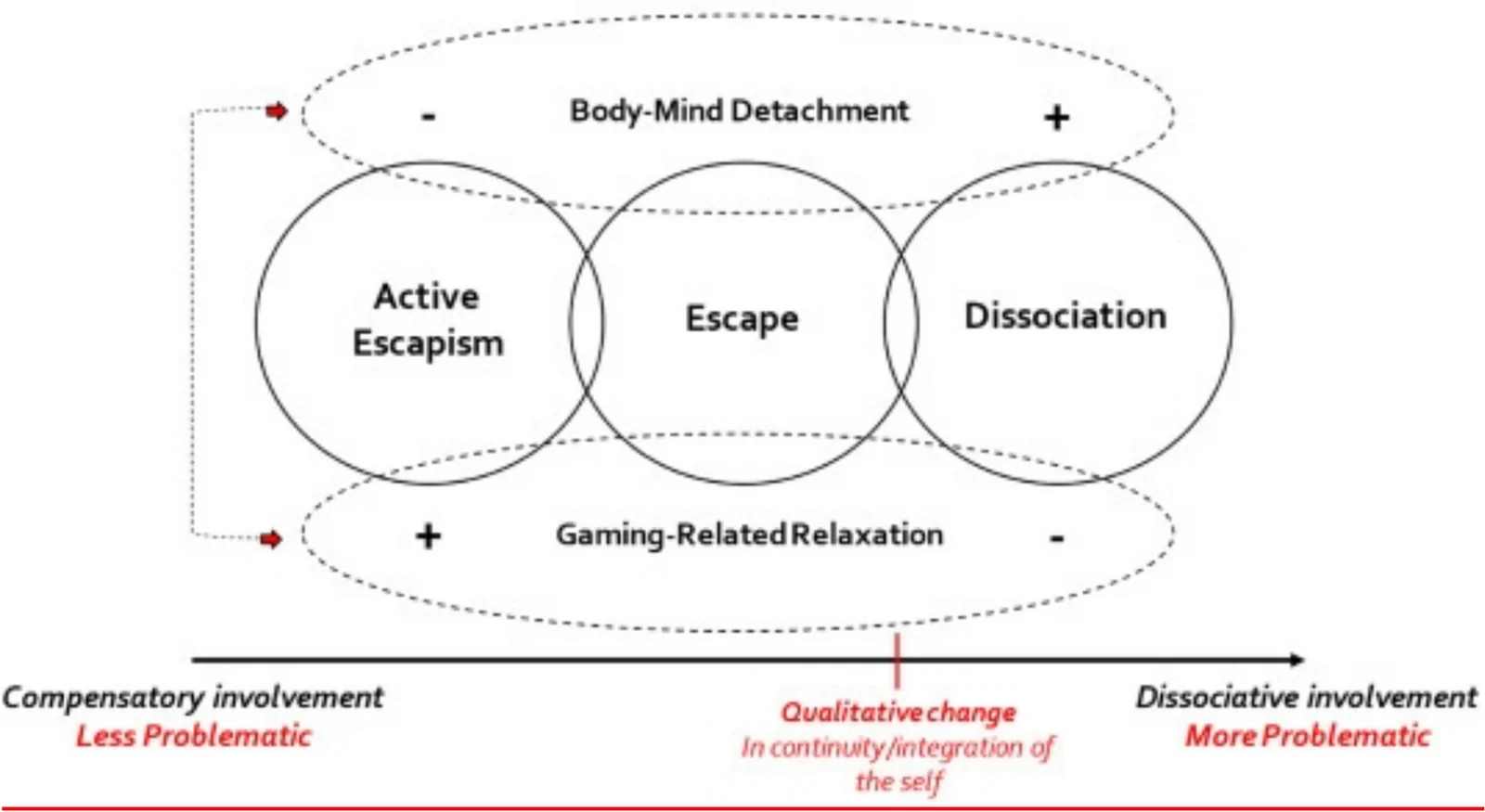
The Compensatory-Dissociative Online Gaming model (Giardina et al., 2024a)

Movement along this continuum is operationalized through three core processes: active escapism, escape, and dissociation. Active escapism reflects a bidirectional use of the virtual environment to simulate and process real life stressors, with the expectation of returning to reality with adaptive insights. Escape represents an intermediate process characterized by withdrawal from a perceived intolerable physical world, in which virtual engagement functions as a refuge rather than a compensatory resource. Dissociation marks the most severe endpoint, involving a pronounced disconnection between environments and self representations aimed at preventing emotional disintegration (Fig. 1).

In the present study, this framework is extended from online gaming to pornography use. Pornographic environments are conceptualized as immersive virtual spaces that similarly enable emotion regulation, identity related processes, and need compensation. Along the compensatory end of the continuum, pornography use may temporarily address unmet needs while remaining integrated with offline relational and sexual experiences. As involvement shifts toward escape and dissociation, pornography use increasingly reflects avoidance and compartmentalization, with reduced integration of bodily, relational, and self-related aspects of sexuality. This adapted C-DOG framework enhances conceptual specificity and provides a coherent basis for distinguishing compensatory from more problematic patterns of pornography consumption.

The current study employed a person-centered approach to identify distinct profiles among young adults exhibiting varying degrees of compensatory and dissociative tendencies, grounded in the compensatory dissociative model of emotional and behavioral self-regulation (Giardina et al., 2024a, 2024b). Rather than treating compensatory-dissociative involvement as a homogeneous construct, this approach captures how these tendencies intersect with behavioral patterns at the individual level. By focusing on intra-individual configurations, the analysis allows for the detection of psychologically meaningful subgroups shaped by different constellations of risk and resilience factors (Collins & Lanza, 2010; Hofmans et al., 2020), thus offering a more nuanced understanding of how compensatory and dissociative dynamics manifest across individuals.

Compensatory and dissociative tendencies were examined as core constructions because of their established relevance following psychological trauma and their role in shaping maladaptive coping strategies (Giardina et al., 2024a; Schimmenti, 2018). Prior research has linked problematic pornography use and pornography consumption with psychopathological symptoms (Ince et al., 2021), impulsivity (Bocci Benucci et al., 2024), loneliness (Mestre-Bach & Potenza, 2023a, 2023b), emotion regulation difficulties (Testa et al., 2024), and frustration or satisfaction of basic psychological needs (Bőthe et al., 2020). Although previous studies have explored the connection between early childhood trauma and dissociative processes (Dutra et al., 2009; Silberg, 2021), the current study extends this literature by integrating early experiences within a compensatory and dissociative framework. Problematic pornography use has similarly been viewed as a maladaptive strategy to manage emotional distress, regain control, or distance oneself from unresolved trauma (Gewirtz-Meydan, 2024; Wéry et al., 2019), highlighting the theoretical importance of this model for understanding self-regulation and behavioral risks during emerging adulthood.

### Childhood Maltreatment

Childhood maltreatment represents a major global concern with far reaching consequences for development and well being. It refers to a range of harmful experiences, including physical, emotional, and sexual abuse, as well as physical and emotional neglect, all of which can undermine children’s psychological and relational functioning (Gardner et al., 2019; Krug et al., 2002; Melamed et al., 2024). International estimates indicate that approximately one third of children worldwide are exposed to emotional abuse, over one fifth to physical abuse, and nearly one sixth to neglect (World Health Organization, 2014).

Despite this broad scope, research examining links between childhood maltreatment and problematic pornography use has tended to focus narrowly on childhood sexual abuse, often overlooking other forms of abuse and neglect (Bergeron et al., 2022; Gewirtz-Meydan, 2024). This limited focus obscures the cumulative and interactive effects of diverse maltreatment experiences. Taken together, exposure to sexual, physical, and emotional abuse, as well as neglect, constitutes a substantial public health challenge worldwide and underscores the need for more integrative approaches when examining later maladaptive coping behaviors, including problematic pornography use (Wéry et al., 2019).

### Problematic Pornography Use

Problematic pornography use has gained growing attention as a prevalent expression of compulsive sexual behavior, marked by persistent difficulties in regulating consumption despite associated distress or interference with daily functioning (Ince et al., 2026; Kraus et al., 2018). Epidemiological evidence suggests that approximately 2 to 10 percent of young adults may exhibit elevated risk for this pattern of use (Bőthe et al., 2023, 2024b). Higher vulnerability has been associated with frequent masturbation and heightened involvement in sex related cognitive and affective processes, including intrusive thoughts, sexual fantasies, and physiological arousal (Bőthe et al., 2021).

Accumulating research indicates that, for a subset of individuals, problematic pornography use is accompanied by a range of negative outcomes (McGraw et al., 2024; Vaillancourt Morel & Bergeron, 2019; Wizła & Lewczuk, 2024). These include increased symptoms of depression and anxiety, disruptions in intimacy and romantic relationships (Twohig et al., 2009; Mestre Bach & Potenza, 2023a), as well as occupational and financial difficulties (Schneider, 2000). Additionally, problematic use has been associated with complex and mixed links to erectile dysfunction (Glica et al., 2023; Grubbs & Gola, 2019) and with an elevated risk of HIV and other sexually transmitted infections (Scanavino et al., 2023; Yoon et al., 2016), underscoring its relevance as a public health concern (Bőthe et al., 2022).

### The Current Study

Given the complexity of compensatory and dissociative processes and the limited application of this framework to pornography use, the present study was designed as an exploratory, person-centered investigation. The study aimed to identify naturally occurring profiles of compensatory and dissociative tendencies and to examine how these profiles differ across theoretically relevant psychological, behavioral, and developmental domains. Based on these frameworks, several theoretically informed expectations guided the analyses. Profiles characterized by stronger dissociative tendencies were expected to show higher levels of psychological distress, emotion regulation difficulties, and problematic pornography use, whereas profiles marked by more compensatory tendencies were expected to show higher engagement levels with comparatively better psychological functioning. Childhood maltreatment, particularly emotional and sexual forms, was expected to differentiate profiles and to shape variations in pornography use and problematic use across profiles.

## Method

### Participants

The sample consisted of 608 Israeli young adults recruited from diverse regions across Israel. Recruitment was open to individuals of all genders and religious affiliations; however, the final sample included 436 men (73%) and 165 women (27%), with ages ranging from 18 to 27 years (M = 24.3, SD = 4.8). Most participants (92%) identified as Israeli. Regarding socioeconomic status, participants primarily rated themselves as having good (71%) or very good (21%) status, with smaller proportions reporting bad (7.1%) or very bad (0.5%). With respect to religious affiliation, 14% identified as religious, 29% as traditional, 1.8% as ultra orthodox, and 55% as secular.

### Measures

*Problematic Pornography Consumption Scale* (PPCS; Bőthe et al., 2021). The PPCS consists of six items assessing problematic pornography use over the past six months (e.g., “When I promise myself that I will stop watching porn, I can only stop for a short time”). Each item represents one of Griffiths’ (2005) addiction components: salience, tolerance, mood modification, relapse, withdrawal, and conflict. Items are rated on a 7-point Likert scale (1 = never to 7 = all the time). Higher scores indicate greater problematic use. The scale was translated and validated in Hebrew (Bőthe et al., 2023). Internal consistency was excellent (Cronbach’s α = .89).

*Frequency of Pornography Consumption.* Pornography use frequency was assessed using a single item that measured the frequency of engagement in this behavior. Participants responded to the question “Do you watch pornography?” on an 11-point scale ranging from 0 (not at all) to 11 (more than seven times per week).

*Childhood Trauma Questionnaire–Short Form* (CTQ-SF; Bernstein et al., 1998). The CTQ-SF is a 28-item retrospective self-report questionnaire that assesses childhood traumatic experiences across five facets: emotional abuse, physical abuse, sexual abuse, emotional neglect, and physical neglect. Each facet consists of five items (e.g., “I was hit with hard objects” for physical abuse). Responses use a 5-point Likert scale (1 = never true to 5 = very often true). The scale was administered in its Hebrew version (Efrati, 2023). Reliability was good to excellent for all facets: emotional abuse (α = .85), physical abuse (α = .85), sexual abuse (α = .92), emotional neglect (α = .87), and physical neglect (α = .65). Mean scores were calculated for each facet.

*Revised UCLA Loneliness Scale*–Version 3 (Russell, 1996; Russell et al., 1980). This 20-item instrument measures feelings of loneliness and social isolation (e.g., “There is no one I can turn to”). Participants rate statements on a 4-point scale (1 = never to 4 = often). The scale was administered in its Hebrew version (Efrati & Amichai-Hamburger, 2019). Higher total scores indicate greater loneliness. Internal consistency was excellent (Cronbach’s α = .93).

*Short UPPS-P Impulsivity Scale* (Cyders et al., 2014). This 20-item measure assesses five dimensions of impulsivity: positive urgency, negative urgency, lack of perseverance, lack of premeditation, and sensation seeking. Responses were recorded on a 4-point Likert scale (1 = strongly agree to 4 = strongly disagree), with items reverse-scored so higher scores indicate greater impulsivity. The scale was administered in its Hebrew version (Efrati & Spada, 2024). Cronbach’s α values ranged from .73 to .78.

*16-item Difficulties in Emotion Regulation Scale* (DERS-16; Bjureberg et al., 2016). The DERS-16 assesses emotion regulation difficulties across five dimensions: non-acceptance, goals, impulse control, strategies, and clarity. Items are rated on a 5-point Likert scale (1 = almost never to 5 = almost always). The scale was administered in its Hebrew version (Pat-Horenczyk et al., 2012). Internal consistency was excellent for the total scale (α = .93), and acceptable to good for subscales: non-acceptance (α = .86), goals (α = .83), impulsivity (α = .90), strategies (α = .90), and clarity (α = .85).

*Depression, Anxiety, and Stress Scales*—21-item version (DASS-21; Lovibond & Lovibond, 1995). The DASS-21 measures the severity of depression, anxiety, and stress symptoms over the past week (e.g., “I felt that I had nothing to look forward to”). Items are rated on a 4-point Likert scale (0 = does not apply to me at all to 3 = applies to me very much or most of the time). The scale was administered in its Hebrew version (Efrati, 2023). Subscale internal consistency was excellent: depression (α = .90), anxiety (α = .85), and stress (α = .89). Subscale scores were calculated individually.

*Basic Psychological Need Satisfaction and Frustration Scale* (BPNSFS; Chen et al., 2015). The BPNSFS assesses satisfaction and frustration with psychological needs encompassing autonomy, competence, and relatedness, each with satisfaction and frustration subscales. (e.g., “I feel that the people I care about also care about me”). Items are rated on a 5-point Likert scale (1 = completely untrue to 5 = completely true). The scale was translated and validated in Hebrew (Grubbs et al., 2025). Cronbach’s α values for satisfaction subscales were autonomy (α = .82), competence (α = .84), and relatedness (α = .83); for frustration subscales: autonomy (α = .73), competence (α = .71), and relatedness (α = .85). Scores were calculated separately for satisfaction and frustration scales.

*Compensatory-Dissociative Pornographic Consumption Scale* (C-DPCS) is a 36-item measure adapted from the C-DOG scale (Giardina et al., 2024b) for use in the context of pornography consumption in the present study. The 36-item C-DPCS assesses compensatory-dissociative patterns of pornography consumption over the past 12 months. It measures six factors: emotional displacement (using pornography to reduce tension), absorption (detachment during pornography use), active escapism (simulative use to compensate for the lack of self-confidence to achieve things in real life), virtual withdrawal (use linked to impaired social functioning), dissociative regulation (use driven by anxiety), and failure escape (use motivated by fear of failure). Although the factor labels of the C-DPCS are derived from the original C-DOG framework, it is important to note that the empirical structure of the scale does not fully mirror the theoretical components of the C-DOG model. As shown in Giardina et al. (2024b), several compensatory processes, including active escapism, function as cross cutting mechanisms across multiple factors, and some empirically derived dimensions such as virtual withdrawal extend beyond the initially hypothesized theoretical structure. Items are rated on a 7-point Likert scale (1 = never to 7 = all the time). Although based on the C-DOG framework, the C-DPCS represents an adaptation rather than a fully validated standalone scale. The scale was translated into Hebrew and subsequently back translated into English to ensure linguistic equivalence. The Hebrew version demonstrated excellent internal consistency: emotional displacement (α = .85), absorption (α = .86), active escapism (α = .85), dissociative regulation (α = .94), virtual withdrawal (α = .93), and failure escape (α = .89).

To appraise the factorial structure of the C-DPCS in the present sample, we conducted a confirmatory factor analysis specifying six correlated latent factors corresponding to the theorized subscales. Given the ordinal nature of the items, the model was estimated using a diagonally weighted least squares (DWLS/WLSMV-type) estimator, treating all items as ordered categorical indicators. The analysis provided strong support for the proposed six-factor measurement model. Global model fit was excellent (CFI = .998; TLI = .998; RMSEA = .043, 90% CI [.040, .047]; SRMR = .047). All items loaded significantly and strongly on their intended latent factors, with standardized loadings ranging from .80 to .98, indicating high convergent validity at the item level. Explained variance was substantial across indicators (R2 values predominantly exceeding .65), further supporting the adequacy of the factor–item mappings. Correlations were high (approximately .66-.90), consistent with prior work showing that compensatory-dissociative mechanisms are highly interrelated and may share a strong common core, while remaining empirically distinguishable. To examine whether the high correlations warrant collapsing subscales, we compared a higher-order model—in which a single second-order general factor accounts for the covariances among the six first-order factors—with a unidimensional model. The higher-order model fit the data well (CFI = .983; TLI = .982; RMSEA = .051 [90% CI .047, .054]; SRMR = .052), supporting the interpretation that the subscales reflect distinguishable facets of a common underlying compensatory-dissociative dimension. The unidimensional model showed poorer fit (CFI = .979; TLI = .978; RMSEA = .056 [90% CI .053, .060]; SRMR = .056). These results indicate that collapsing the six subscales into a single scale result in loss of fit, and that retaining separate subscales under a higher-order structure better represents the data.

On this basis, we retained the six subscales for the latent profile analysis, as this approach preserves the distinct variance that differentiated profiles in theoretically meaningful ways (see Fig. 3). Overall, these results indicate that the Hebrew adaptation of the C-DPCS exhibits a well-defined and empirically supported factorial structure in the context of pornography consumption.

### Procedure

Participants were young adults aged 18–27 from diverse regions across Israel, inclusive of all genders and religious affiliations. Recruitment strategies included posting announcements on university and community bulletin boards and engaging potential participants via social media platforms, including Instagram and TikTok. The questionnaire was specifically distributed to a non-clinical population. Individuals currently receiving clinical care or diagnosed with persistent mental health conditions were not directly excluded via screening questions; rather, our recruitment strategy targeted only the general (non-clinical) community, implicitly excluding clinical populations. Eligible participants were informed that the study aimed to examine patterns of pornography consumption among Israeli young adults. After providing informed consent, participants completed an anonymous online questionnaire via the Polyeda survey platform. The measures were presented in random order, each accompanied by clear instructions. Participants were instructed to complete the questionnaire privately in a quiet, distraction-free environment. Of the 806 individuals who were contacted, 608 completed the questionnaire, yielding a response rate of 75.4%. Completion of the survey took approximately 14 min. All measures were administered in Hebrew, the native language of most participants. 

### Data Analysis

The analyses were conducted on 608 participants. Before the primary analyses, we examined the normal distribution of all main study measures using a series of Anderson–Darling normality tests. We also assessed the presence of multivariate outliers using the Minimum Covariance Determinant approach (performed with the *MASS* R package). We found that all measures significantly deviated from normality (all *A* > 2.18, all *p* < 1.53^−5^ or lower). In addition, 182 observations were identified as multivariate outliers. Because these observations were assumed to reflect genuine individual differences rather than data errors, they were retained in the dataset and their influence was addressed by employing robust statistical methods throughout (e.g., trimmed means, heteroscedastic ANOVAs, and robust regression), which are specifically designed to limit the impact of multivariate outliers without excluding cases. Additionally, missing data were permitted, resulting in 6.98% of the data being absent, with 28 distinct patterns. Jamshidian and Jalal’s non-parametric Missing Completely At Random (MCAR) test indicated that the data were missing at random (MAR; Hawkins’ *χ*^*2*^_(12)median_ = 106.56, *p*_median_ = 2.86^−17^, Anderson–Darling *T*_median_ = 8.89, *p*_median_ = .023). Therefore, we addressed missing data using the Multiple Imputation procedure (Rubin, 2009) with seven complete sets in keeping with the percentage of missing data, which were implemented using the *mice* and *micemd* R packages. The reported results represent the pooled outcome of the MI procedure.

**Fig. 2:**
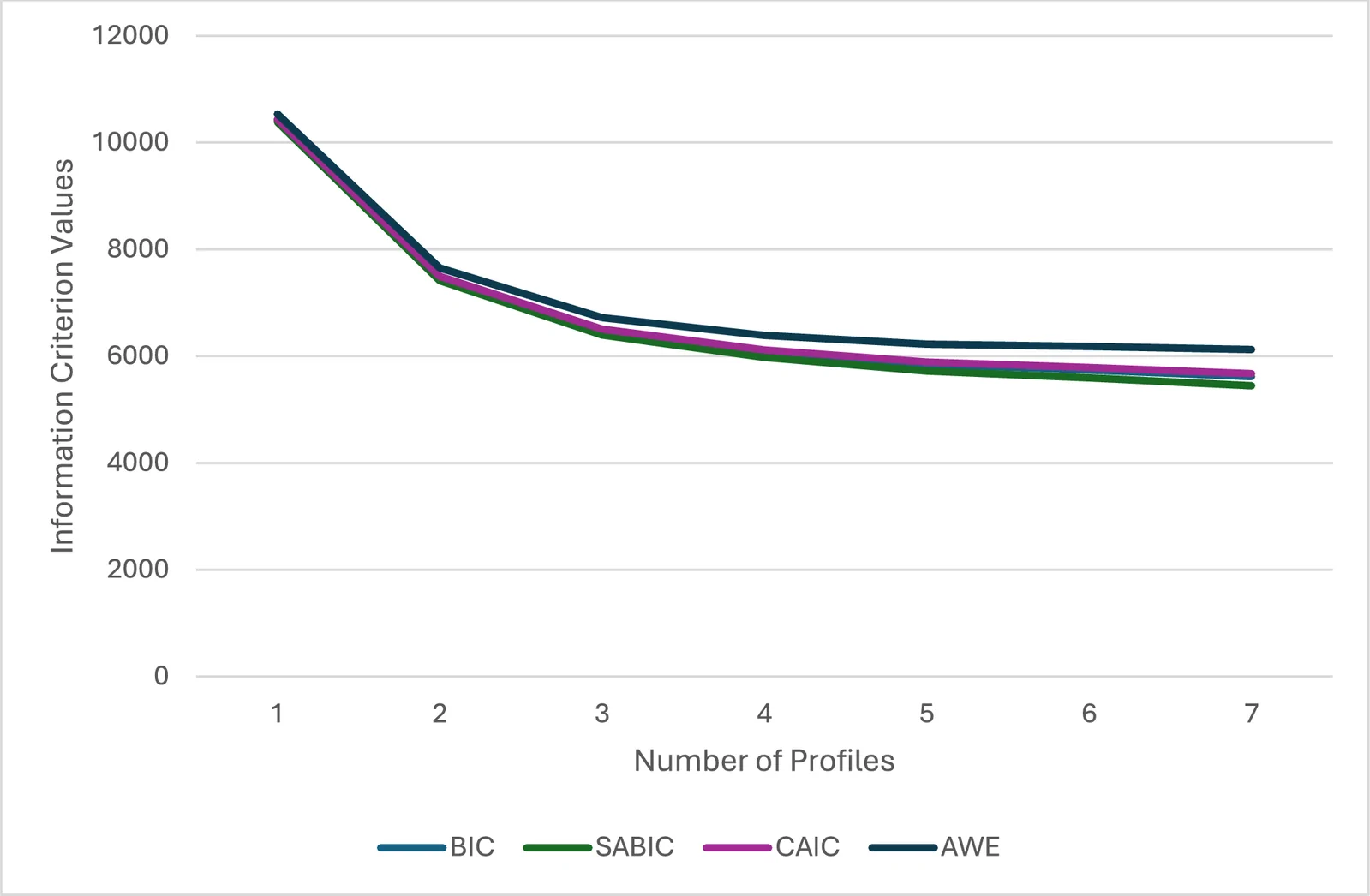
Information criteria scree plot

We began by applying latent profile analysis to estimate distinct latent profiles in participants’ compensatory-dissociative clusters (emotional displacement, absorption, active escapism, dissociative regulation, virtual withdrawal, and failure to escape). To do this, we follow the guidelines of Nylund-Gibson and Choi (2018) using the *tidyLPA* R package with MPlus 8.8 (Muthén & Muthén, 2019) Structural Equation Modeling integration. We examined one to seven possible profiles using unconditional LPA. To decide on the number of profiles, we used the following information (summarized in Table 1): (1) information criteria (IC)—including the Bayesian Information Criterion (BIC), sample-size adjusted Bayesian Information Criterion (SABIC), Consistent Akaike Information Criterion (CAIC), and Approximate Weight of Evidence Criterion (AWE)—which are approximate fit indices where lower values indicate superior fit. These ICs were also plotted (see Fig. 2) to inspect for an “elbow” of point of “diminishing returns” in model fit (equivalent to a scree plot in factor analysis). (2) We also used the bootstrapped likelihood ratio test (BLRT), which provides *p*-values to assess whether adding a class leads to a statistically significant improvement in model fit. The BLRT is one of the most robust methods across various modeling conditions (Nylund et al., 2007). (3) We considered the Bayes Factor (BF) indices for pairwise comparisons of fit between two adjacent class models, with values greater than 10 suggesting strong support for the more complex model. Additionally, we assessed the correct model probability (cmP), which estimates the likelihood of each model being “correct” among all models evaluated. We also considered how the selected models relate to each other (e.g., theoretical differences) as well as the relative sizes of the emergent classes. Here, we established a minimum profile size of 60 participants, representing 10% of the sample.

**Table 1:** Fit indices for 1 to 7 profile solutions

	1 profile	2 profiles	3 profiles	4 profiles	5 profiles	6 profiles	7 profiles
BIC	10,423.49	7474.08	6478.00	6080.88	5849.47	5740.69	**5617.16**
SABIC	10,385.40	7413.76	6395.46	5976.11	5722.48	5591.48	**5445.73**
CAIC	10,435.49	7493.08	6504.00	6113.88	5889.47	5787.69	**5671.16**
AWE	10,534.41	7650.90	6720.75	6389.49	6224.00	6181.08	**6123.41**
BLRT		2994.29	1040.95	441.99	276.29	153.65	**168.40**
BF		1.11 × 10^64^	4.26 × 10^21^	4.20 × 10^8^	1.06 × 10^5^	230.15	**481.28**
cmP	0.00	0.00	0.00	0.00	0.00	0.00	**1.00**
Entropy	1	0.987	0.957	0.966	0.938	0.946	0.952
LL	− 5173.29	− 3676.14	− 3155.67	− 2934.67	− 2796.53	− 2719.71	− 2635.51
% smallest n		0.16	0.13	0.03	0.03	0.03	0.02

BIC = Bayesian Information Criterion; SABIC = Sample-size adjusted Bayesian Information Criterion; CAIC = Consistent Akaike Information Criterion; AWE = Approximate Weight of Evidence Criterion; BLRT = bootstrapped likelihood ratio test; BF = Bayes Factor; cmP = correct model probability. Bolded values reflect the best fit as reflected by the index. A minimum profile size of 60 participants equals 10.0% or higher

In the next phase, we examined differences between profiles in several areas: (1) early childhood trauma (emotional, physical, and sexual abuse, and physical and emotional neglect); (2) psychopathological symptoms (depression, anxiety, and stress); (3) impulsivity (negative urgency, lack of perseverance, lack of premeditation, sensation seeking, and positive urgency); (4) loneliness; (5) difficulties in emotion regulation (non-acceptance of emotional responses, difficulties engaging in goal-directed behavior, impulse control difficulties, limited access to emotional regulation strategies, and lack of emotional clarity), (6) basic psychological needs (autonomy satisfaction and frustration, relatedness satisfaction and frustration, and competence satisfaction and frustration), and (7) problematic pornography consumption and pornography consumption frequency. To assess these differences, we conducted a series of one-way analyses of variance on trimmed means under the assumption of homoscedasticity, followed by Yuen’s test for trimmed means with Holm correction for post-hoc comparisons. All *p*-values were adjusted using a false discovery rate (FDR) of 5% to account for multiple comparisons.

In the final part of the results, we tested whether participants’ early childhood trauma moderated the association between profile membership and two outcomes: (1) problematic pornography consumption, and (2) participants’ pornography consumption frequency. To this end, we conducted a series of robust regression analyses using the *robustbase* R package, modeling the interactions between profile membership and the cluster of early childhood trauma as predictors of each outcome. All trauma subscales were mean-centered prior to computing interaction terms to facilitate interpretation and reduce multicollinearity. To appraise the stability of the results, we conducted the analyses twice, with and without controlling for the following measures: General Severity Index (total score of DASS), loneliness, difficulties in emotion regulation, sensation seeking, satisfaction, and frustration (the latter two are composite scores of the basic psychological needs questionnaire). Where significant interactions emerged, we conducted simple slopes analyses using the *interactions* R package to probe the nature of the effects.

## Results

### Latent Profile Analysis

**Fig. 3:**
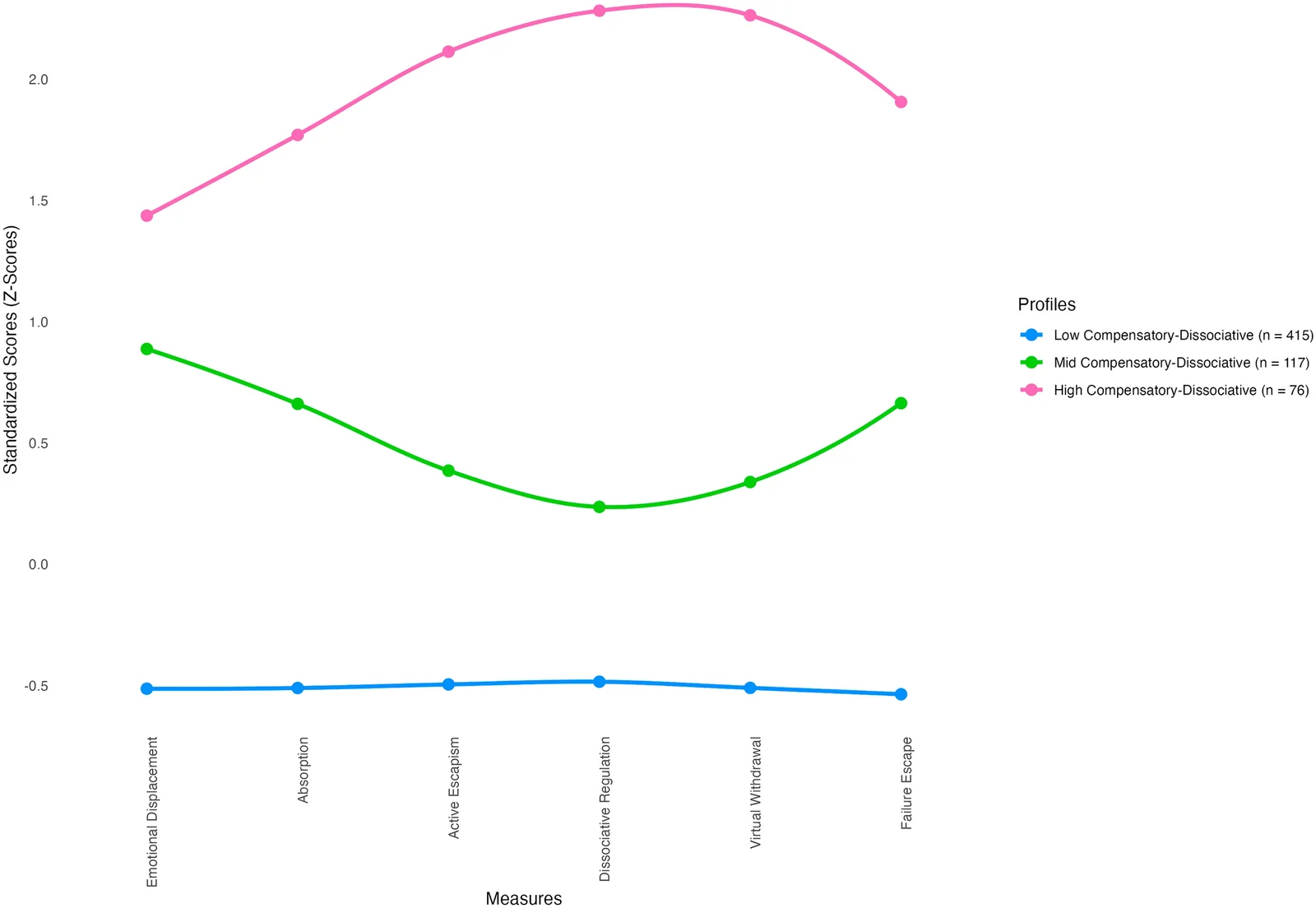
The selected 3-profile solution. The measures used in the LPA are presented on the x-axis. Relative scores are presented on the y-axis

The results are summarized in Table 1. As is often the case in applied research (Nylund-Gibson & Choi, 2018), the fit indices did not converge on a single optimal solution. The scree plot of information criteria (Fig. 2) reveals a noticeable “elbow” above the three-profile solution, suggesting its relative superiority. The cmP, BF, and BLRT favored the seven-profile solution. However, the seven-profile solution included four profiles with fewer participants than our recommended threshold, consisting of 43 (7.01%), 21 (3.45%), 21 (3.45%), and 14 (2.30%) participants. Consistent with best-practice recommendations (Nylund-Gibson & Choi, 2018; Spurk et al., 2020), final model selection was based on a balance between statistical fit and substantive considerations, including class size, stability, and interpretability. Considering all fit indices and sample size concerns, we selected the three-profile solution. This model offered well-differentiated profiles, adequate sample sizes within each group, and was supported by multiple information criteria. The entropy score of 0.96 indicates exceptionally clear separation between profiles (Nagin & Nagin, 2005). The profiles are presented in Fig. 3 and include the following groups: Low Compensatory-Dissociative (n = 415; 68.26%), Mid Compensatory-Dissociative (n = 117; 19.24%), and High Compensatory-Dissociative (n = 76; 12.50%).

Participants in the Low Compensatory-Dissociative profile exhibited low levels (average z = − .5) across the emotional displacement, absorption, active escapism, dissociative regulation, virtual withdrawal, and failure escape clusters. The remaining two profiles were characterized by distinct patterns of compensatory-dissociative clusters. Participants in the Mid Compensatory-Dissociative profile reported elevated levels of emotional displacement and failure to escape as compared with absorption, active escapism, dissociative regulation, and virtual withdrawal. Conversely, participants of the High Compensatory-Dissociative profile showed a mirror pattern: diminished levels of emotional displacement and failure escape as compared with absorption, active escapism, dissociative regulation, and virtual withdrawal.

### Differences Between Profiles in the Main Study Measures

As shown in Table 2, significant differences emerged across profiles on the following measures: emotional and physical abuse and neglect; depression, anxiety, and stress; negative urgency, lack of perseverance and premeditation, and positive urgency; difficulties in emotion regulation; all domains of basic psychological needs (autonomy satisfaction and frustration, relatedness satisfaction and frustration, and competence satisfaction and frustration); and both problematic pornography use and pornography consumption frequency. These differences remained significant after applying a 5% FDR correction. Effect sizes ranged from small to large, indicating meaningful variability in the magnitude of between-profile differences across domains.

**Table 2:** Comparisons between profiles in the main study measures

Characteristic	Low Compensatory-Dissociative N = 415	Mid Compensatory-Dissociative N = 117	High Compensatory-Dissociative N = 76	q-value	ξ	Post-hoc
Emotional Abuse	8.58 (4.34)	10.21 (5.23)	11.50 (5.17)	< 0.001	0.34	L < M, H
Physical Abuse	6.10 (2.69)	6.79 (3.72)	8.18 (4.76)	0.014	0.35	L < H
Sexual Abuse	6.49 (3.87)	6.69 (3.88)	8.70 (6.07)	0.12	0.34	–
Physical Neglect	6.62 (2.45)	6.93 (2.36)	8.85 (3.92)	< 0.001	0.41	L, M < H
Emotional Neglect	9.40 (4.36)	10.14 (4.50)	11.03 (4.35)	0.004	0.25	L < H
Depression Symptoms	0.76 (0.69)	1.09 (0.81)	1.40 (0.79)	< 0.001	0.42	L < M < H
Anxiety Symptoms	0.45 (0.53)	0.63 (0.65)	1.07 (0.73)	< 0.001	0.51	L < M < H
Stress Levels	0.93 (0.69)	1.13 (0.77)	1.54 (0.72)	< 0.001	0.43	L < M < H
Negative Urgency	1.97 (0.75)	2.26 (0.73)	2.53 (0.79)	< 0.001	0.37	L < M < H
Lack of Perseverance	1.92 (0.63)	2.03 (0.66)	2.14 (0.79)	0.10	0.18	–
Lack of Premeditation	1.81 (0.61)	1.82 (0.58)	2.17 (0.76)	< 0.001	0.32	L, M < H
Sensation Seeking	2.40 (0.73)	2.34 (0.75)	2.52 (0.78)	0.20	0.16	–
Positive Urgency	1.74 (0.66)	2.06 (0.67)	2.47 (0.74)	< 0.001	0.53	L < M < H
Loneliness	2.73 (0.27)	2.80 (0.28)	2.72 (0.30)	0.052	0.16	L < M
DERS Total Score	31.37 (16.76)	37.45 (17.31)	47.72 (14.23)	< 0.001	0.50	L < M < H
Autonomy Satisfaction	3.81 (0.83)	3.58 (0.81)	3.11 (0.84)	< 0.001	0.48	L > M > H
Autonomy Frustration	2.84 (0.87)	3.06 (0.78)	3.35 (0.88)	< 0.001	0.31	L < M < H
Relatedness Satisfaction	4.18 (0.80)	4.05 (0.72)	3.33 (0.79)	< 0.001	0.53	L > M > H
Relatedness Frustration	1.95 (0.77)	2.32 (0.85)	3.06 (0.79)	< 0.001	0.62	L < M < H
Competence Satisfaction	4.12 (0.76)	3.91 (0.79)	3.35 (0.87)	< 0.001	0.48	L > M > H
Competence Frustration	2.06 (0.97)	2.56 (1.04)	2.94 (0.83)	< 0.001	0.48	L < M < H
PPU	1.65 (0.69)	3.35 (0.90)	4.26 (1.31)	< 0.001	0.85	L < M < H
Pornography Prevalence	5.16 (3.10)	8.35 (1.99)	7.85 (3.13)	< 0.001	0.59	L < M, H

L = Low Compensatory-Dissociative; M = Mid Compensatory-Dissociative; H = High Compensatory-Dissociative

Post-hoc pairwise comparisons conducted using Yuen’s robust *t*-test (20% trimming) with FDR correction. "—" indicates no significant pairwise differences

ξ = explanatory measure of effect size from robust ANOVA

The differences followed a consistent pattern: participants in the High Compensatory-Dissociative profile reported the most adverse outcomes, whereas those in the Low Compensatory-Dissociative profile exhibited the most favorable outcomes. The strongest effects were observed for problematic pornography use and pornography consumption frequency (large effects), followed by difficulties in emotion regulation and relatedness frustration (moderate-to-large effects), whereas symptom severity, impulsivity-related traits, and childhood trauma indicators generally showed small-to-moderate effects. The only exception was pornography consumption frequency, which was highest among participants in the Mid Compensatory-Dissociative profile rather than the High profile.

### Differences Between Profiles in Problematic Pornography Use and Pornography Consumption Frequency as a Function of Early Childhood Trauma

Results are presented in Table 3 and Supplementary Table 1. Analyses revealed significant main effects of childhood sexual abuse: Greater severity was associated with both higher levels of problematic pornography use and increased frequency of pornography consumption. Additionally, five significant interactions emerged between profile membership and early childhood trauma.

**Table 3:** Robust regressions for predicting problematic pornography use and pornography consumption frequency by participants’ profile and early childhood trauma

	Problematic Pornography Use			Pornography Frequency		
Predictors	Estimates	*CI*	*p*	Estimates	*CI*	*p*
(Intercept)	1.60	1.53 – 1.67	** < 0.001**	5.17	4.87 – 5.48	** < 0.001**
Low vs. Mid Compensatory-Dissociative	1.73	1.57 – 1.88	** < 0.001**	3.22	2.58 – 3.87	** < 0.001**
Low vs. High Compensatory-Dissociative	2.45	2.24 – 2.65	** < 0.001**	2.58	1.73 – 3.43	** < 0.001**
Emotional Abuse	− 0.01	− 0.03 – 0.02	0.532	0.05	− 0.05 – 0.16	0.327
Physical Abuse	− 0.03	− 0.06 – 0.01	0.125	− 0.04	− 0.18 – 0.10	0.597
Sexual Abuse	0.03	0.01 – 0.05	**0.006**	0.10	0.01 – 0.18	**0.023**
Physical Neglect	0.01	− 0.03 – 0.05	0.560	− 0.11	− 0.27 – 0.05	0.191
Emotional Neglect	0.01	− 0.01 – 0.04	0.235	0.08	− 0.03 – 0.18	0.144
Low vs. Mid × Emotional Abuse	− 0.02	− 0.06 – 0.03	0.506	− 0.11	− 0.31 – 0.08	0.242
Low vs. High × Emotional Abuse	0.12	0.06 – 0.18	** < 0.001**	0.20	− 0.05 – 0.45	0.118
Low vs. Mid × Physical Abuse	0.03	− 0.03 – 0.09	0.306	0.03	− 0.23 – 0.28	0.847
Low vs. High × Physical Abuse	0.06	− 0.02 – 0.13	0.124	0.16	− 0.14 – 0.46	0.300
Low vs. Mid × Sexual Abuse	− 0.02	− 0.07 – 0.02	0.249	− 0.07	− 0.25 – 0.11	0.449
Low vs. High × Sexual Abuse	− 0.05	− 0.09 – − 0.01	**0.010**	− 0.29	− 0.45 – − 0.12	**0.001**
Low vs. Mid × Physical Neglect	0.01	− 0.07 – 0.09	0.720	0.03	− 0.31 – 0.37	0.853
Low vs. High × Physical Neglect	0.06	− 0.03 – 0.15	0.164	0.19	− 0.16 – 0.55	0.286
Low vs. Mid × Emotional Neglect	0.02	− 0.03 – 0.07	0.439	0.05	− 0.16 – 0.27	0.637
Low vs. High × Emotional Neglect	− 0.11	− 0.17 – − 0.05	** < 0.001**	− 0.33	− 0.57 – − 0.09	**0.008**
Observations	608			608		
R^2^ / R^2^ adjusted	0.675 / 0.666			0.230 / 0.208		

**Fig. 4:**
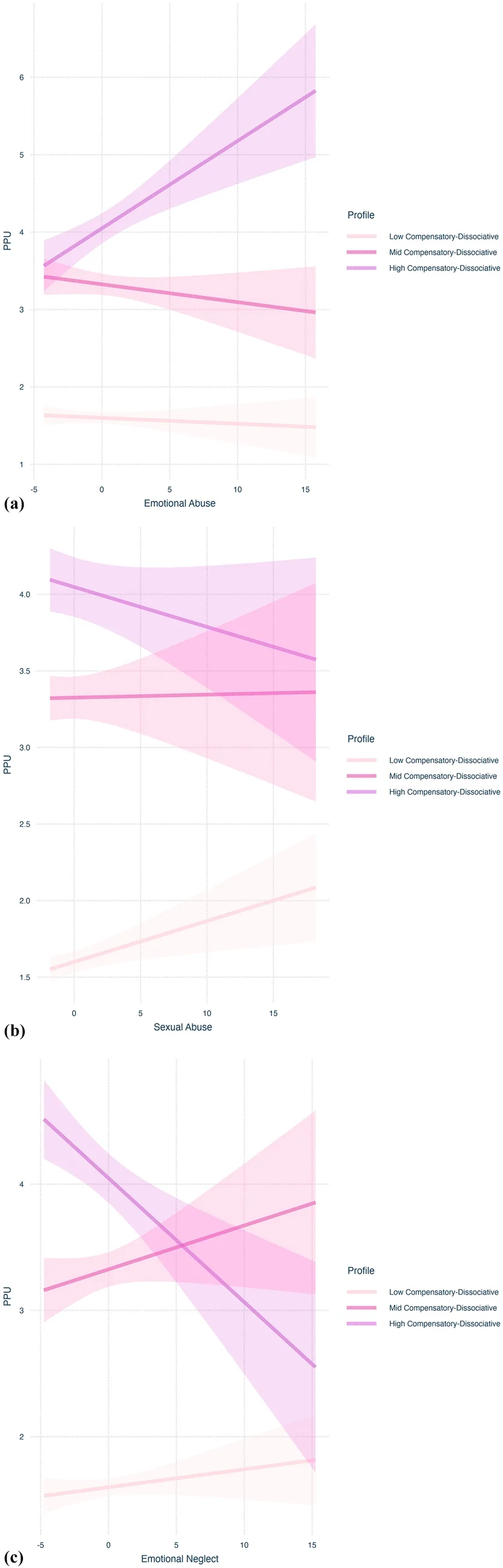
**a** Interaction between profile and emotional abuse in predicting participants’ problematic pornography use. **b** Interaction between profile and sexual abuse in predicting participants’ problematic pornography use. **c** Interaction between profile and emotional neglect in predicting participants’ problematic pornography use

Specifically, emotional abuse, sexual abuse, and emotional neglect moderated the relationship between profile membership and problematic pornography use. For emotional abuse, a significant positive association with problematic pornography use was found only among participants in the High Compensatory-Dissociative profile (b = 0.11, SE = .03, t = 4.03, p < .001; see Fig. 4a). This relationship was not significant for the Mid (b = -0.02, SE = .02, t = − 1.17, p = .24) or Low (b = − 0.01, SE = .01, t = − 0.62, p = .53) profiles.

Different patterns were observed for sexual abuse (Fig. 4b) and emotional neglect (Fig. 4c). In the Mid (b = 0.00, SE = .02, *t* = 0.10, *p* = .92) and High (b = − 0.03, SE = .02, *t* = -1.44, *p* = .15) profiles, problematic pornography use remained elevated regardless of the severity of sexual abuse. In contrast, for the Low Compensatory-Dissociative profile, higher severity of sexual abuse was significantly associated with increased problematic pornography use (b = 0.03, SE = .01, *t* = 2.78, *p* < .01).

Emotional neglect showed an opposite pattern to emotional abuse. Among participants in the High profile, greater emotional neglect was associated with lower levels of problematic pornography use (b = -0.10, SE = .03, *t* = -3.65, *p* < .001). This association was not significant in the Mid (b = 0.03, SE = .02, *t* = 1.47, *p* = .14) or Low (b = 0.01, SE = .01, *t* = 1.19, *p* = .23) profiles.

**Fig. 5:**
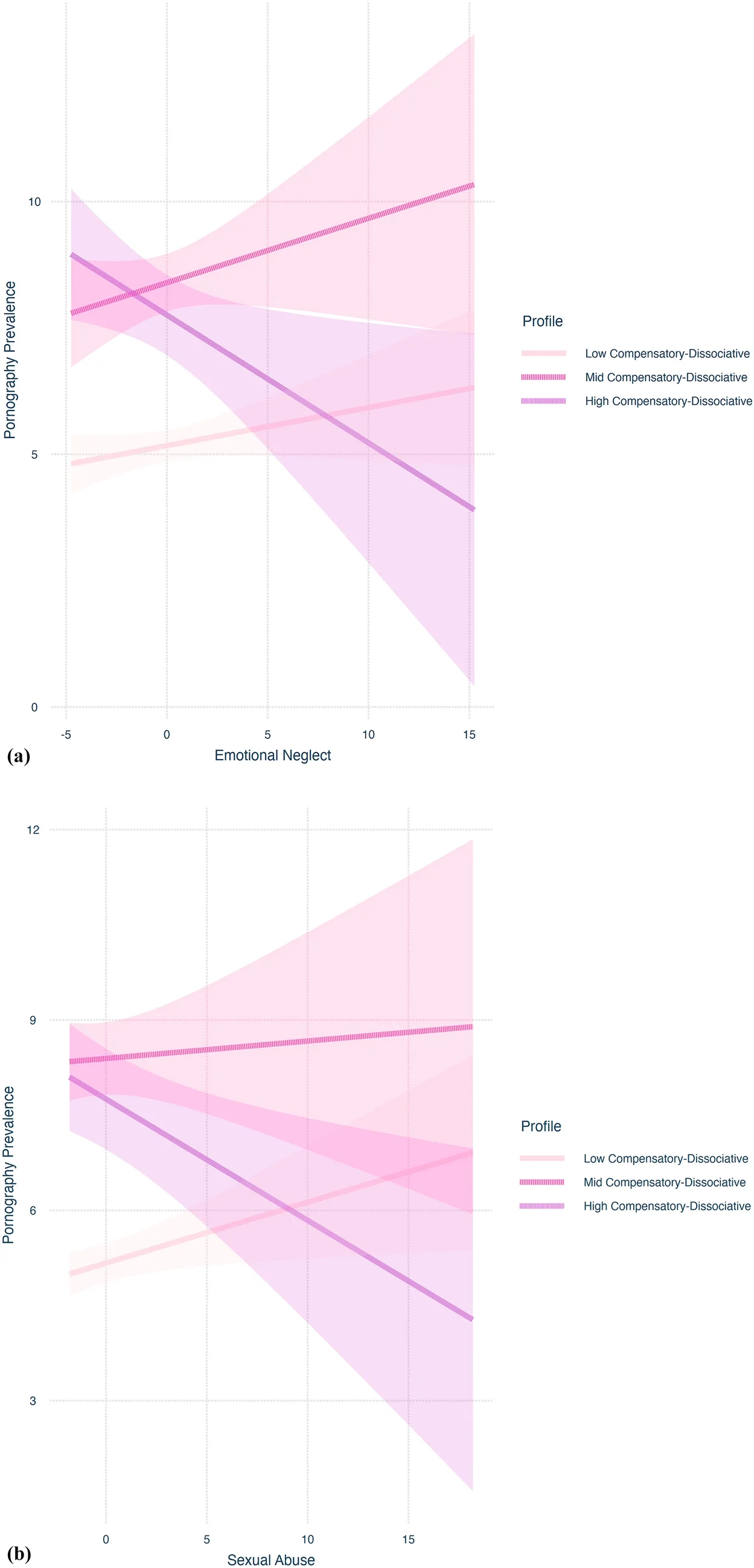
**a** Interaction between profile and sexual abuse in predicting participants’ pornography consumption frequency. **b** Interaction between profile and emotional neglect in predicting participants’ pornography consumption frequency

Sexual abuse (Fig. 5a) and emotional neglect (Fig. 5b) also moderated the relationship between profile membership and frequency of pornography consumption. In the High profile, greater severity of sexual abuse was associated with lower frequency of use (b = -0.19, SE = .07, *t* = − 2.62, *p* < .01). Conversely, in the Low profile, higher severity predicted greater frequency of pornography consumption (b = 0.10, SE = .04, *t* = 2.28, *p* = .02).

A similar pattern emerged for emotional neglect: Only in the High profile did greater severity correspond to reduced frequency of pornography use (b = − 0.25, SE = .11, *t* = − 2.25, *p* = .02). This association was not significant in the Mid (b = 0.13, SE = .10, *t* = 1.31, *p* = .19) or Low (b = 0.08, SE = .05, *t* = 1.46, *p* = .14) profiles.

## Discussion

The present study aimed to identify distinct young adult profiles based on compensatory and dissociative tendencies and to examine their associations with early childhood trauma, psychopathological symptoms, impulsivity, loneliness, emotion regulation difficulties, satisfaction and frustration of basic psychological needs, PPU, and pornography consumption frequency. In this framework, compensatory pornography use refers to engagement aimed at managing stress or unmet needs while remaining psychologically connected to everyday functioning, whereas dissociative use reflects engagement characterized by detachment, emotional numbing, or withdrawal from reality. The findings align with prior research indicating that compensatory and dissociative tendencies differentiate between intensive yet nonproblematic behaviors and maladaptive patterns (Giardina et al., 2024a). The present study extends this framework to pornography use, highlighting the role of these processes in shaping patterns of engagement.

Taken together, the findings indicate that young adults can be meaningfully differentiated into three compensatory-dissociative profiles that reflect graded differences in psychological functioning and pornography-related behaviors. Individuals in the High Compensatory-Dissociative profile exhibited the most adverse psychological outcomes, whereas those in the Low profile demonstrated the most adaptive functioning. Importantly, childhood trauma, particularly emotional abuse, sexual abuse, and emotional neglect, did not exert uniform effects but interacted with individuals’ coping profiles, revealing distinct pathways through which trauma relates to problematic pornography use and consumption frequency. These findings underscore the central role of compensatory and dissociative coping styles in shaping how trauma is translated into behavioral outcomes.

The study identified three distinct profiles of compensatory-dissociative coping among young adults, reflecting varying psychological responses to distress. Individuals in the Low profile showed minimal reliance on either compensatory or dissociative strategies. In contrast, the Mid profile was characterized by elevated emotional displacement and failure escape, reflecting a compensatory pattern of pornography use aimed at managing unmet needs and coping with fear of failure (Vieira et al., 2025; Wang et al., 2024). The High profile, by contrast, was characterized by stronger dissociative tendencies, including absorption and psychological withdrawal, suggesting that pornography use in this group may function less as a tool for emotional regulation and more as a means of detachment (Engel et al., 2023). These findings support the compensatory-to-dissociative continuum proposed in the C-DOG model (Giardina et al., 2024a, 2024b) and extend their relevance to patterns of pornography use.

Across all psychological, emotional, and behavioral measures, individuals in the High Compensatory-Dissociative profile showed the most adverse outcomes, while those in the Low profile exhibited the most adaptive functioning. Notably, frequency of pornography use peaked in the Mid profile—consistent with literature suggesting that compensatory, rather than dissociative, mechanisms drive higher engagement with pornography as a form of emotional regulation (Bőthe et al., 2024a).

An additional moderation analysis revealed a theoretically coherent yet counterintuitive pattern linking childhood trauma and pornography use across compensatory-dissociative profiles. Individuals in the High Compensatory-Dissociative profile exhibited the most adverse psychological functioning overall, yet paradoxically reported lower levels of pornography use despite experiencing greater sexual abuse and emotional neglect. This unexpected finding becomes clearer when examining this group’s distinct dissociative coping style: They exhibited elevated levels of absorption, active escapism, dissociative regulation, and virtual withdrawal, alongside lower emotional displacement and reduced reliance on pornography to cope with fear of failure in real life.

These dissociative traits suggest a reliance on sophisticated internal coping mechanisms that may serve as alternative pathways for managing trauma-related distress (Dalenberg et al., 2012), thereby reducing the need for external compensatory behaviors such as pornography use. Indeed, prior research demonstrates that dissociation during sexual experiences is associated with sexual dysfunction, impaired intimacy, and emotional detachment (Gewirtz-Meydan & Godbout, 2023; Schimmenti & Caretti, 2016). For individuals in the High profile group, pornography may not only be unnecessary—it may be fundamentally incompatible with their preferred coping mode, which centers on psychological disengagement rather than stimulation or arousal (Fernandez et al., 2021). Another possible explanation is that although pornography use was lower, this profile, characterized by a dissociative state, may be associated with other unexamined risk behaviors such as risky sexual activity (Gewirtz-Meydan & Godbout, 2023) or suicidality (Brokke et al., 2022). Future research should investigate these additional behavioral risks to better understand the broader implications of this profile.

In contrast, participants in the Low Compensatory-Dissociative profile group, who lacked such dissociative capacities, demonstrated the expected pattern: Higher trauma exposure was associated with greater problematic pornography use and consumption. For these individuals, pornography likely functions as a compensatory behavior aimed at downregulating emotional arousal or fulfilling unmet attachment needs stemming from their traumatic experiences (Efrati & Amichai-Hamburger, 2019; Efrati et al., 2026; Wang et al., 2024).

Beyond sexual abuse, the present findings underscore the central and differentiated roles of emotional abuse and emotional neglect in shaping how compensatory and dissociative profiles relate to problematic pornography use and consumption frequency. Emotional abuse emerged as a significant moderator only within the High Compensatory-Dissociative profile, where greater exposure was associated with higher levels of problematic pornography use. This pattern suggests that emotionally abusive environments, characterized by chronic criticism, humiliation, or emotional threat, may amplify dysregulated sexual coping specifically among individuals who already rely on dissociative regulatory strategies (Ince et al., 2023; Lew-Starowicz et al., 2020). In such cases, pornography use may serve as a means of managing persistent negative self-representations and heightened emotional arousal rooted in emotionally abusive experiences.

In contrast, emotional neglect showed an inverse moderating pattern within the same High profile. Greater emotional neglect was associated with lower levels of problematic pornography use and reduced frequency of consumption. Although counterintuitive, this finding aligns with theoretical and clinical accounts suggesting that prolonged emotional unavailability may foster emotional numbing, interpersonal withdrawal, and diminished engagement with embodied or relational aspects of sexuality (Gewirtz-Meydan et al., 2026; Malkemus et al., 2025). For individuals characterized by strong dissociative tendencies, severe emotional neglect may therefore reduce motivation for externally oriented compensatory behaviors such as pornography use, even in the presence of elevated psychological distress. This interpretation is consistent with prior work linking emotional neglect to dissociative disengagement, sexual detachment, and reduced sexual responsiveness rather than heightened sexual seeking (Gewirtz-Meydan et al., 2026).

Importantly, these moderation effects were absent in the Mid and Low profiles, highlighting that emotional abuse and neglect exert their influence on pornography-related outcomes primarily when embedded within a broader dissociative coping configuration. This pattern reinforces the value of a process-oriented approach, suggesting that the same traumatic experiences may lead to markedly different behavioral outcomes depending on individuals’ dominant self-regulatory strategies (Kuhl & Quirin, 2025; Posner & Rothbart, 2000).

Notably, physical abuse and physical neglect did not moderate the associations between profile membership and pornography outcomes. One possible explanation is that these forms of maltreatment, while undeniably harmful, may exert more generalized effects on psychopathology (Jaffee, 2017) rather than directly shaping sexual or dissociative coping pathways. Another possibility is that limited variability or lower prevalence of severe physical maltreatment within this community-based sample reduced statistical power to detect moderation effects. Additionally, emotional forms of maltreatment may be more proximally linked to internal working models of the self, affect regulation, and relational schemas, which are particularly relevant to the development of compensatory and dissociative engagement with sexual content.

These findings highlight how the type of available coping strategy, particularly the degree of reliance on dissociation, can fundamentally shape how trauma manifests in behavior. Rather than assuming a uniform effect of trauma on pornography use, the data suggest that individual coping profiles moderate this relationship, resulting in divergent pathways of adaptation or maladaptation. This moderating effect underscores the importance of considering individual differences in coping mechanisms when examining trauma-behavior relationships.

### Limitations and Directions for Future Research

Several limitations of this study should be acknowledged. First, the cross-sectional nature of the design precludes conclusions about causal relationships or developmental trajectories; future longitudinal research is needed to clarify temporal patterns. Second, although data were collected through anonymous self-report surveys, the possibility of socially desirable responding cannot be ruled out, particularly given the sensitivity of the subject matter. Participants may have underreported behaviors such as pornography use or psychological distress due to feelings of shame, fear of judgment, or internalized cultural and moral norms. Third, although the sample was relatively large and included participants with varying levels of religiosity, it was limited to young adults in Israel and consisted only of men and women. In addition, sexual orientation and broader gender identities were not assessed. Given that cultural norms and sociocultural contexts shape both pornography use and its meanings, future research should examine the generalizability of these findings across more diverse populations and cross-cultural settings. Fourth, this study focused on pornography-related behaviors as indicators of self-regulation but did not capture the broader spectrum of sexual expression in young adults. While problematic pornography use and consumption are important dimensions, they represent only part of the picture. Future research should expand the scope to include additional facets such as sexual risk-taking, assertiveness, and experiences of coercion to provide a more comprehensive view of sexual functioning. Furthermore, other psychological processes—such as social comparison, perceptions of pornography’s realism, and potential bidirectional relationships—may also influence the link between compensatory-dissociative profiles and pornography use and should be explored in subsequent studies. Fifth, the sample was predominantly male, which may have introduced gender-related bias, as men typically report higher levels of pornography use and problematic use and may differ from women in trauma-related sexual coping patterns (Nagy et al., 2025). Sixth, the C-DPCS is an early-stage adaptation of the C-DOG scale developed for the present study and should not yet be considered a fully validated measure; accordingly, findings involving this scale should be interpreted cautiously and replicated in future validation research.

Finally, a conceptual limitation concerns the adaptation of the C-DOG scale to pornography use. Although the C-DPCS captures compensatory and dissociative processes, its empirically derived factors do not fully align with the original components of the C-DOG theoretical model. In particular, the Active Escapism dimension may not translate straightforwardly to pornography use, which lacks the interactive and agentic features of gaming. This mismatch may help explain why elevated Active Escapism characterized the High rather than the Mid profile, suggesting that escapism in pornography use may be less adaptive and more closely intertwined with dissociative processes.

### Clinical Implications

The findings highlight the importance of adopting a profile-sensitive approach when assessing and treating young adults with problematic pornography-related behaviors. Rather than viewing the impact of childhood maltreatment as uniform, clinicians should consider the distinct patterns of compensatory and dissociative coping that individuals may exhibit. Recognizing these varied psychological configurations allows for more precise risk assessment and supports the creation of targeted, personalized interventions that address the underlying self-regulatory processes contributing to problematic pornography use and consumption.

Furthermore, the results suggest that compensatory-dissociative profiles may function as transdiagnostic vulnerability factors, linking childhood maltreatment to maladaptive sexual behaviors through shared regulatory mechanisms. Interventions that strengthen adaptive emotion regulation and reduce reliance on dissociative strategies may thus have broad clinical utility. Preventive programs should also incorporate media literacy and promote healthy sexual development to foster more integrated, resilient coping strategies for young adults.

### Conclusions

This study identified distinct compensatory-dissociative profiles among young adults, each associated with unique patterns of trauma exposure, psychological functioning, and pornography-related behaviors. The findings underscore that the way individuals cope—especially their reliance on dissociative strategies—shapes how trauma influences behavior. Rather than a uniform response, trauma-related outcomes appear to be moderated by coping style, highlighting the need for personalized, developmentally informed interventions and future research that accounts for individual regulatory profiles.

## Supplementary Information

Below is the link to the electronic supplementary material.

Supplementary file1 (DOCX 22 KB)

Supplementary file2 (DOCX 26 KB)

## References

[CR1] Bergeron, S., Bigras, N., & Vaillancourt-Morel, M. P. (2022). Child maltreatment and couples’ sexual health: A systematic review. *Sexual Medicine Reviews*, *10*, 567–582. 10.1016/j.sxmr.2022.04.002 36028437

[CR2] Bernstein, D. P., Fink, L., Handelsman, L., & Foote, J. (1998). *Childhood Trauma Questionnaire (CTQ).* APA PsycTests. 10.1037/t02080-000

[CR3] Bjureberg, J., Ljótsson, B., Tull, M. T., Hedman, E., Sahlin, H., Lundh, L. G., & Gratz, K. L. (2016). Development and validation of a brief version of the Difficulties in Emotion Regulation Scale: The DERS-16. *Journal of Psychopathology and Behavioral Assessment*, *38*, 284–296.‏ 10.1007/s10862-015-9514-x PMC4882111 27239096

[CR4] Bocci Benucci, S., Di Gesto, C., Ghinassi, S., Casale, S., & Fioravanti, G. (2024). Pornography use, problematic pornography use, impulsivity, and sensation seeking: A meta-analysis. *Journal of Sexual Medicine,**21*(10), 922–939. 39183145 10.1093/jsxmed/qdae101

[CR8] Bőthe, B., Koós, M., Nagy, L., Kraus, S. W., Demetrovics, Z., Potenza, M. N., & Vaillancourt-Morel, M. P. (2023). Compulsive sexual behavior disorder in 42 countries: Insights from the International Sex Survey and introduction of standardized assessment tools. *Journal of Behavioral Addictions, 12*(2), 393–407. 10.1556/2006.2023.00028 PMC10316175 37352095

[CR10] Bőthe, B., Nagy, L., Koós, M., Demetrovics, Z., Potenza, M. N., International Sex Survey Consortium, & Van Hout, M. C. (2024b). Problematic pornography use across countries, genders, and sexual orientations: Insights from the international sex survey and comparison of different assessment tools. *Addiction*, *119*(5), 928–950. 10.1111/add.16431 38413365

[CR6] Bőthe, B., Tóth-Király, I., Bella, N., Potenza, M. N., Demetrovics, Z., & Orosz, G. (2021). Why do people watch pornography? The motivational basis of pornography use. *Psychology of Addictive Behaviors,**35*(2), 172–186. 10.1037/adb0000603 32730047

[CR5] Bőthe, B., Tóth-Király, I., Potenza, M. N., Orosz, G., & Demetrovics, Z. (2020). High-frequency pornography use may not always be problematic. *Journal of Sexual Medicine,**17*(4), 793–811. 32033863 10.1016/j.jsxm.2020.01.007

[CR7] Bőthe, B., Vaillancourt-Morel, M. P., & Bergeron, S. (2022). Associations between pornography use frequency, pornography use motivations, and sexual wellbeing in couples. *Journal of Sex Research,**59*(4), 457–471. 33724108 10.1080/00224499.2021.1893261

[CR9] Bőthe, B., Vaillancourt-Morel, M.-P., Bergeron, S., Hermann, Z., Ivaskevics, K., Kraus, S. W., Grubbs, J. B., & Problematic Pornography Use Machine Learning Study Consortium. (2024a). Uncovering the most robust predictors of problematic pornography use: A large-scale machine learning study across 16 countries. *Journal of Psychopathology and Clinical Science, 133*(6), 489–502. 10.1037/abn0000913 38884980

[CR11] Brokke, S. S., Bertelsen, T. B., Landrø, N. I., & Haaland, V. Ø. (2022). The effect of sexual abuse and dissociation on suicide attempt. *BMC Psychiatry,**22*(1), 29 10.1186/s12888-021-03662-9. 35012509 PMC8751353

[CR12] Calleja, G. (2010). Digital games and escapism. *Games and Culture,**5*(4), 335–353.

[CR13] Chen, B., Vansteenkiste, M., Beyers, W., Boone, L., Deci, E. L., Van der Kaap-Deeder, J., Duriez, B., Lens, W., Matos, L., Mouratidis, A., Ryan, R. M., Sheldon, K. M., Soenens, B., Van Petegem, S., & Verstuyf, J. (2015). Basic psychological need satisfaction, need frustration, and need strength across four cultures. *Motivation and Emotion,**39*(2), 216–236. 10.1007/s11031-014-9450-1

[CR14] Collins, L. M., & Lanza, S. T. (2010). *Latent class and latent transition analysis: With applications in the social, behavioral, and health sciences*. Wiley.

[CR15] Coulson, M., Oskis, A., Spencer, R., & Gould, R. L. (2020). Tourism, migration, and the exodus to virtual worlds: Place attachment in massively multiplayer online gamers. *Psychology of Popular Media,**9*(4), 525–532. 10.1037/ppm0000244

[CR16] Cyders, M. A., Littlefield, A. K., Coffey, S., & Karyadi, K. A. (2014). Examination of a short English version of the UPPS-P impulsive behavior scale. *Addictive Behaviors,**39*(9), 1372–1376. 24636739 10.1016/j.addbeh.2014.02.013 PMC4055534

[CR17] Dalenberg, C. J., Brand, B. L., Gleaves, D. H., Dorahy, M. J., Loewenstein, R. J., Cardena, E., & Spiegel, D. (2012). Evaluation of the evidence for the trauma and fantasy models of dissociation. *Psychological Bulletin*, *138*(3), 550–588. 10.1037/a0027447 22409505

[CR18] Dutra, L., Bureau, J. F., Holmes, B., Lyubchik, A., & Lyons-Ruth, K. (2009). Quality of early care and childhood trauma: A prospective study of developmental pathways to dissociation. *Journal of Nervous and Mental Disease,**197*(6), 383–390. 19525736 10.1097/NMD.0b013e3181a653b7 PMC2697443

[CR19] Efrati, Y. (2023). Risk and protective factor profiles predict addictive behavior among adolescents. *Comprehensive Psychiatry,**123*, Article 152387. 37037172 10.1016/j.comppsych.2023.152387

[CR20] Efrati, Y., & Amichai-Hamburger, Y. (2019). The use of online pornography as compensation for loneliness and lack of social ties among Israeli adolescents. *Psychological Reports,**122*(5), 1865–1882. 30185120 10.1177/0033294118797580

[CR22] Efrati, Y., Shmulewitz, D., Skvirsky, V., Vider, M., Kor, A., Lev-Ran, S., & Mikulincer, M. (2026). Longitudinal study of probable compulsive sexual behavior disorder and problematic pornography use profiles: Their prospective impact on psychopathology during wartime. *Addictive Behaviors,**175*, Article 108589. 41435755 10.1016/j.addbeh.2025.108589

[CR21] Efrati, Y., & Spada, M. M. (2024). Development and validation of the metacognitions about sex scale: Exploring its role as a mediator between negative affect, emotion dysregulation strategies, and compulsive sexual behavior disorder. *Journal of Sex & Marital Therapy,**50*(1), 76–93. 37878755 10.1080/0092623X.2023.2259894

[CR23] Engel, J., Carstensen, M., Veit, M., Sinke, C., Kneer, J., Hartmann, U., & Kruger, T. H. (2023). Personality dimensions of compulsive sexual behavior in the Sex@ Brain study. *Journal of Behavioral Addictions,**12*(2), 408–420. 37384566 10.1556/2006.2023.00029 PMC10316167

[CR24] Fernandez, D. P., Kuss, D. J., & Griffiths, M. D. (2021). The pornography “rebooting” experience: A qualitative analysis of abstinence journals on an online pornography abstinence forum. *Archives of Sexual Behavior,**50*(2), 711–728. 33403533 10.1007/s10508-020-01858-w PMC7889567

[CR70] Gardner, M. J., Thomas, H. J., & Erskine, H. E. (2019). The association between five forms of child maltreatment and depressive and anxiety disorders: A systematic review and meta-analysis. *Child Abuse & Neglect, 96*, 104082. 10.1016/j.chiabu.2019.104082 31374447

[CR25] Gewirtz-Meydan, A. (2024). Traumatized sexuality: Understanding and predicting profiles of sexual behaviors using childhood abuse and trauma measures. *Child Maltreatment,**29*(2), 350–363. 36583251 10.1177/10775595221148425

[CR26] Gewirtz-Meydan, A., & Godbout, N. (2023). Between pleasure, guilt, and dissociation: How trauma unfolds in the sexuality of childhood sexual abuse survivors. *Child Abuse & Neglect,**141*, Article 106195. 37116448 10.1016/j.chiabu.2023.106195

[CR27] Gewirtz-Meydan, A., Spivak-Lavi, Z., Kraus, S. W., Vaillancourt-Morel, M. P., Nagy, L., Bergeron, S., & ISS Consortium. (2026). A network analysis of embodied trauma: Self-destructive behaviors among survivors of childhood and adolescent/adult sexual assault. *Journal of Psychology*, *160*, 516–543 10.1080/00223980.2025.2594534 41424167

[CR30] Giardina, A., Fournier, L., Starcevic, V., King, D. L., Di Blasi, M., Schimmenti, A., & Billieux, J. (2024b). From active escapism to virtual withdrawal: Validation of the Compensatory-Dissociative Online Gaming scales (C-DOGs). *Journal of Behavioral Addictions,**13*(4), 1028–1050. 39601788 10.1556/2006.2024.00059 PMC11737414

[CR29] Giardina, A., Schimmenti, A., Starcevic, V., King, D. L., Di Blasi, M., & Billieux, J. (2024a). Problematic gaming, social withdrawal, and escapism: The Compensatory-Dissociative Online Gaming (C-DOG) model. *Computers in Human Behavior,**155*, Article 108187.

[CR31] Glica, A., Wizła, M., Gola, M., & Lewczuk, K. (2023). Hypo-or hyperfunction? Differential relationships between compulsive sexual behavior disorder facets and sexual health. *Journal of Sexual Medicine,**20*(3), 332–345. 36763943 10.1093/jsxmed/qdac035

[CR33] Grubbs, J. B., & Gola, M. (2019). Is pornography use related to erectile functioning? Results from cross-sectional and latent growth curve analyses. *Journal of Sexual Medicine,**16*(1), 111–125. 30621919 10.1016/j.jsxm.2018.11.004

[CR32] Grubbs, J. B., Tóth-Király, I., Nagy, L., Koós, M., Kraus, S. W., Demetrovics, Z., & Bőthe, B. (2025). Basic psychological needs satisfaction: An international examination of invariance across 22 languages and 32 countries. *Motivation and Emotion*, *49*(5), 552–574.‏

[CR34] Hofmans, J., Wille, B., & Schreurs, B. (2020). Person-centered methods in vocational research. *Journal of Vocational Behavior,**118*, Article 103398.

[CR37] Ince, C., Antons, S., Ashton, S., Borgogna, N. C., Brand, M., Briken, P., Castro-Calvo, J., Chen, L., Coleman, E., Efrati, Y., Fernández, D. P., Fuss, J., Gleason, N., Gola, M., Jennings, T. L., Kowalewska, E., Kraus, S. W., Lewczuk, K., Grubbs, J. B., … Bőthe, B. (2026). Compulsive sexual behavior disorder (CSBD) and problematic pornography use (PPU): A comprehensive, interdisciplinary, and expert-informed narrative review with suggested future directions. *Journal of Behavioral Addictions*, *15*, 32–98. 10.1556/2006.2025.00337 41879870 PMC13132360

[CR36] Ince, C., Fontenelle, L. F., Carter, A., Albertella, L., Tiego, J., Chamberlain, S. R., & Rotaru, K. (2023). Clarifying and extending our understanding of problematic pornography use through descriptions of the lived experience. *Scientific Reports,**13*(1), Article 18193. 37875697 10.1038/s41598-023-45459-8 PMC10598215

[CR35] Ince, C., Yücel, M., Albertella, L., & Fontenelle, L. F. (2021). Exploring the clinical profile of problematic pornography use. *CNS Spectrums,**26*(6), 648–657. 10.1017/S1092852920001686 32690117

[CR38] Jaffee, S. R. (2017). Child maltreatment and risk for psychopathology in childhood and adulthood. *Annual Review of Clinical Psychology,**13*, 525–551. 28375720 10.1146/annurev-clinpsy-032816-045005

[CR39] Kor, A., Djalovski, A., Potenza, M. N., Zagoory-Sharon, O., & Feldman, R. (2022). Alterations in oxytocin and vasopressin in men with problematic pornography use: The role of empathy. *Journal of Behavioral Addictions,**11*(1), 116–127. 35040806 10.1556/2006.2021.00089 PMC9109630

[CR74] Kraus, S. W., Krueger, R. B., Briken, P., First, M. B., Stein, D. J., Kaplan, M. S., Voon, V., Abdo, C. H. N., Grant, J. E., Atalla, E., & Reed, G. M. (2018). Compulsive sexual behaviour disorder in the ICD‐11. *World Psychiatry, 17*(1), 109–110. 10.1002/wps.20499 PMC5775124 29352554

[CR71] Krug, E. G., Mercy, J. A., Dahlberg, L. L., & Zwi, A. B. (2002).The world report on violence and health. *The Lancet, 360*(9339),1083–1088. 10.1016/S0140-6736(02)11133-0 12384003

[CR40] Kuhl, J., & Quirin, M. (2025). Individual differences in self-regulation. *Motivation and action* (pp. 585–645). Springer Nature Switzerland.

[CR75] Lew-Starowicz, M., Lewczuk, K., Nowakowska, I., Kraus, S., & Gola, M. (2020). Compulsive sexual behavior and dysregulation of emotion. *Sexual Medicine Reviews, 8*(2), 191-205. 10.1016/j.sxmr.2019.10.003 31813820

[CR41] Lovibond, P. F., & Lovibond, S. H. (1995). The structure of negative emotional states: Comparison of the Depression Anxiety Stress Scales (DASS) with the Beck Depression and Anxiety Inventories. *Behaviour Research and Therapy,**33*(3), 335–343. 7726811 10.1016/0005-7967(94)00075-u

[CR42] Malkemus, S. A., & Smith, J. F. (2025). Sexual disembodiment: Sexual energy, trauma, and the body. *Journal of Humanistic Psychology,**65*(5), 1137–1162.

[CR43] McGraw, J. S., Grant Weinandy, J. T., Floyd, C. G., Hoagland, C., Kraus, S. W., & Grubbs, J. B. (2024). Problematic pornography use and suicidal thoughts: Results from cross-sectional and longitudinal analyses. *Psychology of Addictive Behaviors,**38*(6), 728–738 10.1037/adb0000996. 38451727

[CR72] Melamed, D. M., Botting, J., Lofthouse, K., Pass, L., & Meiser-Stedman, R. (2024). The relationship between negative self-concept, trauma, and maltreatment in children and adolescents: A meta-analysis. *Clinical Child and Family Psychology Review, 27*(1), 220–234.‏ 10.1007/s10567-024-00472-9 PMC10920440 38386241

[CR44] Mestre-Bach, G., & Potenza, M. N. (2023a). Pornography use, problematic pornography use, and potential impacts on partners and relationships. *Current Addiction Reports,**10*(2), 219–229.

[CR45] Mestre-Bach, G., & Potenza, M. N. (2023b). Loneliness, pornography use, problematic pornography use, and compulsive sexual behavior. *Current Addiction Reports,**10*(4), 664–676.

[CR46] Muthén, B., & Muthén, L. (2019). *Mplus: A general latent variable modeling program*. Muthén & Muthén.

[CR47] Nagin, D. S., & Nagin, D. (2005). *Group-based modeling of development*. Harvard University Press.

[CR48] Nagy, L., Bőthe, B., Kraus, S. W., Demetrovics, Z., & Grubbs, J. B. (2025). Does the sexual nature of trauma moderate the relationship between post-traumatic stress and compulsive sexual behaviors? Investigating trauma experiences in the light of compulsive sexual behavior and problematic pornography use. *Sexual Health & Compulsivity,**32*(4), 331–352.

[CR49] Nylund, K. L., Asparouhov, T., & Muthén, B. O. (2007). Deciding on the number of classes in latent class analysis and growth mixture modeling: A Monte Carlo simulation study. *Structural Equation Modeling: A Multidisciplinary Journal,**14*(4), 535–569.

[CR50] Nylund-Gibson, K., & Choi, A. Y. (2018). Ten frequently asked questions about latent class analysis. *Translational Issues in Psychological Science,**4*(4), 440–461. 10.1037/tps0000176

[CR51] Pal, D., & Arpnikanondt, C. (2024). The sweet escape to metaverse: Exploring escapism, anxiety, and virtual place attachment. *Computers in Human Behavior,**150*, Article 107998.

[CR52] Pat-Horenczyk, R., Achituv, M., Rubenstein, A. K., Khodabakhsh, A., Brom, D., & Chemtob, C. (2012). Growing up under fire: Building resilience in young children and parents exposed to ongoing missile attacks. *Journal of Child & Adolescent Trauma,**5*, 303–314.

[CR53] Posner, M. I., & Rothbart, M. K. (2000). Developing mechanisms of self-regulation. *Development and Psychopathology,**12*(3), 427–441. 11014746 10.1017/s0954579400003096

[CR54] Russell, D. W. (1996). UCLA Loneliness Scale (Version 3): Reliability, validity, and factor structure. *Journal of Personality Assessment,**66*(1), 20–40. 8576833 10.1207/s15327752jpa6601_2

[CR55] Russell, D., Peplau, L. A., & Cutrona, C. E. (1980). The revised UCLA Loneliness Scale: Concurrent and discriminant validity evidence. *Journal of Personality and Social Psychology,**39*(3), 472–480 10.1037/0022-3514.39.3.472. 7431205

[CR56] Scanavino, M. D., Guirado, A. G., Marques, J. M., Amaral, M. L. S. A. D., Messina, B., Reis, S. C. D., Scanavino, M. D. T., Amaral, M. L. S. ’Ad., Barros, V. B., Abdo, C. H. N., Tavares, H., & Parsons, J. T. (2023). Treatment effects and adherence of sexually compulsive men in a randomized controlled trial of psychotherapy and medication. *Journal of Behavioral Addictions,**12*(1), 261–277. 36897612 10.1556/2006.2023.00004 PMC10260211

[CR57] Schimmenti, A. (2018). The trauma factor: Examining the relationships among different types of trauma, dissociation, and psychopathology. *Journal of Trauma & Dissociation,**19*(5), 552–571. 29125800 10.1080/15299732.2017.1402400

[CR58] Schimmenti, A., & Caretti, V. (2016). Linking the overwhelming with the unbearable: Developmental trauma, dissociation, and the disconnected self. *Psychoanalytic Psychology,**33*(1), 106–128 10.1037/a0038019.

[CR59] Schneider, J. P. (2000). A qualitative study of cybersex participants: Gender differences, recovery issues, and implications for therapists. *Sexual Addiction & Compulsivity,**7*(4), 249–278.

[CR60] Silberg, J. L. (2021). *The child survivor: Healing developmental trauma and dissociation*. Routledge.

[CR61] Spurk, D., Hirschi, A., Wang, M., Valero, D., & Kauffeld, S. (2020). Latent profile analysis: A review and “how to” guide of its application within vocational behavior research. *Journal of Vocational Behavior,**120*, Article 103445. 10.1016/j.jvb.2020.103445

[CR62] Testa, G., Villena-Moya, A., & Chiclana-Actis, C. (2024). Emotional dysregulation and coping strategies in the context of problematic pornography use: A narrative review. *Current Addiction Reports,**11*(2), 229–241.

[CR63] Twohig, M. P., Crosby, J. M., & Cox, J. M. (2009). Viewing Internet pornography: For whom is it problematic, how, and why? *Sexual Addiction & Compulsivity,**16*(4), 253–266.

[CR64] Vaillancourt-Morel, M. P., & Bergeron, S. (2019). Self-perceived problematic pornography use: Beyond individual differences and religiosity. *Archives of Sexual Behavior,**48*(2), 437–441. 30116928 10.1007/s10508-018-1292-6

[CR65] Vieira, C., Kuss, D. J., & Griffiths, M. D. (2025). Early maladaptive schemas and online pornography use: A cross-sectional study. *International Journal of Mental Health and Addiction.* 10.1007/s11469-025-01463-9

[CR66] Wang, J., Li, R., Tang, S., & Li, H. (2024). Differentiating anticipatory and consummatory reward processing in problematic pornography use: An event-related potential analysis of reward dynamics. *Computers in Human Behavior,**158*, Article 108277.

[CR67] Wéry, A., Schimmenti, A., Karila, L., & Billieux, J. (2019). Where the mind cannot dare: A case of addictive use of online pornography and its relationship with childhood trauma. *Journal of Sex & Marital Therapy,**45*(2), 114–127. 30015567 10.1080/0092623X.2018.1488324

[CR73] World Health Organization. (2014). *Child maltreatment*. Retrieved from https://www.who.int/docs/default-source/documents/childmaltreatment/child-maltreatment-infographic-en.pdf?sfvrsn=7d798249_2

[CR68] Wizła, M., & Lewczuk, K. (2024). Compulsive sexual behavior disorder and problematic pornography use in the context of social ties. *Current Addiction Reports,**11*(2), 275–286.

[CR69] Yoon, I. S., Houang, S. T., Hirshfield, S., & Downing, M. J., Jr. (2016). Compulsive sexual behavior and HIV/STI risk: A review of current literature. *Current Addiction Reports,**3*(4), 387–399.

