# Supplementary material for: Compensatory and Dissociative Coping in Young Adults: A Person-Centered Analysis of Their Associations with Childhood Maltreatment and Problematic Pornography Use
Source: Arch Sex Behav. 2026 Aug 17;55(6):2643–59. doi: 10.1007/s10508-026-03495-1 (PMC13597624; doi:10.1007/s10508-026-03495-1)
Supplement: Supplementary file 1 [file 10508_2026_3495_MOESM1_ESM.docx]

Compensatory-Dissociative Online Pornography scales (C-DOPs)

Please use the following scale of values to indicate how representative each of the statements is of your experience with online pornography during the last year (last 12 months). There are no good or bad statements. Try to be as transparent as possible regarding your experience.

|  | 1  Not at all representative | 2  Not very representative | 3  Not a little nor very representative | 4  Very representative | 5  Fully representative |
| --- | --- | --- | --- | --- | --- |
| 1. I get so immersed while watching pornography that I do not notice the things happening around me in the offline world. |  |  |  |  |  |
| 2. I watch pornography because it reduces the tension. |  |  |  |  |  |
| 3. I find that I can become so involved while watching pornography that it feels like it is really happening to me. |  |  |  |  |  |
| 4. I have the experience of not being sure whether feelings or experiences happened while watching pornography or in offline life. |  |  |  |  |  |
| 5. I have the experience of remembering the pornography so vividly that I feel like I am reliving it again. |  |  |  |  |  |
| 6. Expanding my social circles by talking about the pornography I watched prevents me from feeling isolated |  |  |  |  |  |
| 7. Experiencing a Experiencing a sense of achievement while watching pornography helps me deal with some failures or disappointments in my personal life. |  |  |  |  |  |
| 8. I get so involved in watching pornography that I lose track of time. |  |  |  |  |  |
| 9. Watching pornography helps me to enter a state of peace. |  |  |  |  |  |
| 10. I can feel as if I am looking at the real world as a pornography consumer. |  |  |  |  |  |
| 11. I experience bodily sensations and movements as I would have experienced them while watching pornography. |  |  |  |  |  |
| 12. My perception of some elements in the real world has changed as a result of my experiences watching pornography (e.g., how I perceive my partner’s appearance after watching pornography). |  |  |  |  |  |
| 13. Enjoying the sense of belonging that comes with being a part of a community of people who watch pornography (e.g., social media groups) prevents me from feeling lonely. |  |  |  |  |  |
| 14. I continue watching pornography to avoid the difficult challenges of my life rather than deal with them directly. |  |  |  |  |  |
| 15. After a prolonged time of watching pornography, it is like I “wake up” and suddenly realize that many hours have passed since I started watching. |  |  |  |  |  |
| 16. Watching pornography helps me to approach my negative emotions (e.g., anger) in a more suitable environment. |  |  |  |  |  |
| 17. I engage in specific pornographic content to “metabolize” (elaborate, process, make sense out of) situations in my real life that are somehow emotionally similar to the pornographic experiences. |  |  |  |  |  |
| 18. I have the experience of feeling as if people, objects, and the world around me are not real while this doesn’t happen to me while I am watching pornography. |  |  |  |  |  |
| 19. I find that confronting issues that arise from watching pornography helps me build confidence to deal with life’s problems. |  |  |  |  |  |
| 20. I resort to watching pornography because I struggle to express who I really am in the real world. |  |  |  |  |  |
| 21. I watch pornography to protect myself from thoughts and emotions related to future challenges and possible failures in social relationships, at school, or in my career. |  |  |  |  |  |
| 22. I sometimes feel like I’m out of my body, watching myself from the outside whilst I am doing something, while this doesn’t happen when I'm watching pornography. |  |  |  |  |  |
| 23. When watching pornography, it happens that I sometimes stop for a brief moment, and suddenly realize that I don’t recognize the room or the world around me, as if they weren’t real. |  |  |  |  |  |
| 24. Through watching pornography, I feel I develop important skills that help me cope with difficulties in achieving my offline life goals. |  |  |  |  |  |
| 25. I watch pornography to avoid feeling judged or rejected by others in offline contexts. |  |  |  |  |  |
| 26. I keep watching pornography so I can postpone doing something challenging I feel I can’t do. |  |  |  |  |  |
| 27. After watching pornography, I feel like my mind has been disconnected from my body. |  |  |  |  |  |
| 28. I identify more with the physical appearance of actors in pornographic films than I identify with my own body. |  |  |  |  |  |
| 29. I prefer watching pornography than having face-to-face social interactions. |  |  |  |  |  |
| 30. I sometimes confuse my own name with the name of a character in a pornographic film. |  |  |  |  |  |
| 31. Watching pornography allows me to stay away from a “toxic” environment (e.g., family quarrels, school bullying, work problems). |  |  |  |  |  |
| 32. When I stop watching pornography, disturbing memories and emotions come to mind. |  |  |  |  |  |
| 33. I feel that watching pornography is a safer place than the offline world. |  |  |  |  |  |
| 34. Intrusive and disturbing images, thoughts or sounds from pornography, come to my mind when I am not watching it. |  |  |  |  |  |
| 35. I find that my experiences from watching pornography are more vivid or memorable than experiences in my real life. |  |  |  |  |  |
| 36. I need to keep I need to keep watching pornography because I do not feel safe when I’m not watching it. |  |  |  |  |  |
