# Supplementary material for: Compensatory and Dissociative Coping in Young Adults: A Person-Centered Analysis of Their Associations with Childhood Maltreatment and Problematic Pornography Use
Source: Arch Sex Behav. 2026 Aug 17;55(6):2643–59. doi: 10.1007/s10508-026-03495-1 (PMC13597624; doi:10.1007/s10508-026-03495-1)
Supplement: Supplementary file 2 [file 10508_2026_3495_MOESM2_ESM.docx]

Supplementary Table 1

Robust Regressions for Predicting PPU and Pornography Consumption Prevalence by Participants’ Profile and Early Childhood Trauma While Controlling for Potential Covariates

|  | **PPU** | | | **Pornography Prevalence** | | |
| --- | --- | --- | --- | --- | --- | --- |
| *Predictors* | *Estimates* | *CI* | *p* | *Estimates* | *CI* | *p* |
| (Intercept) | 0.43 | -0.28 – 1.15 | 0.232 | 0.09 | -2.93 – 3.11 | 0.953 |
| Low vs. Mid Compensatory-Dissociative | 1.67 | 1.52 – 1.82 | **<0.001** | 3.01 | 2.36 – 3.66 | **<0.001** |
| Low vs. High Compensatory-Dissociative | 2.35 | 2.13 – 2.57 | **<0.001** | 2.20 | 1.28 – 3.12 | **<0.001** |
| Emotional Abuse | -0.02 | -0.05 – 0.00 | 0.085 | -0.01 | -0.12 – 0.09 | 0.800 |
| Physical Abuse | -0.01 | -0.05 – 0.02 | 0.379 | -0.01 | -0.15 – 0.13 | 0.887 |
| Sexual Abuse | 0.02 | 0.00 – 0.04 | **0.037** | 0.07 | -0.01 – 0.15 | 0.094 |
| Physical Neglect | 0.02 | -0.02 – 0.06 | 0.309 | -0.07 | -0.23 – 0.09 | 0.385 |
| Emotional Neglect | 0.01 | -0.02 – 0.03 | 0.673 | 0.08 | -0.02 – 0.19 | 0.117 |
| GSI | 0.08 | -0.06 – 0.21 | 0.255 | 0.14 | -0.43 – 0.71 | 0.632 |
| Loneliness | 0.46 | 0.22 – 0.70 | **<0.001** | 1.26 | 0.24 – 2.27 | **0.015** |
| Emotion Regulation | 0.00 | -0.00 – 0.01 | 0.229 | 0.02 | 0.00 – 0.04 | **0.041** |
| Sensation Seeking | 0.12 | 0.04 – 0.21 | **0.003** | 0.24 | -0.11 – 0.59 | 0.175 |
| Satisfaction | -0.11 | -0.21 – -0.01 | **0.038** | 0.04 | -0.40 – 0.48 | 0.852 |
| Frustration | -0.04 | -0.15 – 0.08 | 0.510 | 0.07 | -0.42 – 0.57 | 0.774 |
| Low vs. Mid × Emotional Abuse | -0.02 | -0.06 – 0.03 | 0.481 | -0.08 | -0.27 – 0.11 | 0.384 |
| Low vs. High × Emotional Abuse | 0.11 | 0.05 – 0.17 | **<0.001** | 0.19 | -0.05 – 0.44 | 0.123 |
| Low vs. Mid × Physical Abuse | 0.03 | -0.03 – 0.09 | 0.388 | 0.03 | -0.23 – 0.28 | 0.833 |
| Low vs. High × Physical Abuse | 0.04 | -0.03 – 0.12 | 0.228 | 0.15 | -0.15 – 0.45 | 0.320 |
| Low vs. Mid × Sexual Abuse | -0.02 | -0.06 – 0.02 | 0.353 | -0.04 | -0.22 – 0.13 | 0.642 |
| Low vs. High × Sexual Abuse | -0.04 | -0.08 – -0.00 | **0.048** | -0.27 | -0.43 – -0.10 | **0.001** |
| Low vs. Mid × Physical Neglect | 0.00 | -0.08 – 0.08 | 0.938 | -0.04 | -0.38 – 0.29 | 0.797 |
| Low vs. High × Physical Neglect | 0.05 | -0.04 – 0.13 | 0.301 | 0.14 | -0.22 – 0.50 | 0.446 |
| Low vs. Mid × Emotional Neglect | 0.03 | -0.03 – 0.08 | 0.328 | 0.04 | -0.18 – 0.25 | 0.734 |
| Low vs. High × Emotional Neglect | -0.09 | -0.15 – -0.04 | **0.001** | -0.31 | -0.55 – -0.07 | **0.012** |
| Observations | 608 | | | 608 | | |
| R^2^ / R^2^ adjusted | 0.695 / 0.683 | | | 0.262 / 0.233 | | |
